# Piperlongumine promotes death of retinoblastoma cancer cells

**DOI:** 10.18632/oncotarget.27947

**Published:** 2021-04-27

**Authors:** Nathalie Allaman-Pillet, Daniel F. Schorderet

**Affiliations:** ^1^Institute for Research in Ophthalmology, Sion, Switzerland; ^2^University of Lausanne, Faculty of Biology and Medicine, Lausanne, Switzerland; ^3^Ecole Polytechnique Fédérale de Lausanne, Faculty of Life Sciences, Lausanne, Switzerland

**Keywords:** cancer, retinoblastoma, piperlongumine, programmed cell death

## Abstract

Retinoblastoma is the most common pediatric intraocular malignant tumor. While retinoblastoma initiation is triggered by the inactivation of both alleles of the retinoblastoma tumor suppressor gene (*RB1*) in the developing retina, tumor progression requires additional epigenetic changes, retinoblastoma genomes being quite stable. Although the management of RB has recently improved, new therapeutic agents are necessary to improve the treatment of advanced forms of retinoblastoma.

In this report, we analyzed the pro-death effect of piperlongumine (PL), a natural compound isolated from Piper longum L., on two human retinoblastoma cell lines, WERI-Rb and Y79. The effects of PL on cell proliferation, cell death and cell cycle were investigated. PL effectively inhibited cell growth, impacted the cell cycle by decreasing the level of cyclins and CDK1 and increasing CDKN1A and triggered a caspase-3 independant cell death process in which reactive oxygen species (ROS) production is a major player. Indeed, PL toxicity in retinoblastoma cell lines was inhibited by a ROS scavenger N-acetyl-l-cysteine (NAC) treatment. These findings suggest that PL reduces tumor growth and induces cell death by regulating the cell cycle.

## INTRODUCTION

Retinoblastoma is a malignant tumor derived from photoreceptor precursor cells. It affects retina at a very early stage of childhood with an incidence of one case per 15,000–20,000 live births and represents 4% of all pediatric malignancies [1]. Although the survival rate of patients with retinoblastoma is extremely high in developed countries, left untreated advanced tumors limit eye preservation and expose patients to risks of metastasis and death. Therefore, depending on the disease stage, the therapeutic approaches for retinoblastoma treatment have to be adjusted, including enucleation, intravenous or intra-arterial chemotherapy, or local treatments such as laser therapy, cryotherapy, and radiation [2]. Renuméroter les références.

A multi-step model for the progression of normal retina to retinoblastoma has been proposed [3], the first step being the inactivation of both alleles of the tumor suppressor gene *RB1* in the developing retina. Additional epigenetic modifications following *RB1* inactivation promote subsequent malignant progression [4]. The overall survival in children with RB is related to many factors, such as the tumor size and location. Even if the survival rate of children with RB is high (more than 85%), developing effective therapeutic strategies is the key to significantly improve the overall survival in patients.

Piperlongumine (PL) is a natural alkaloid isolated from long pepper (Piper longum). PL was described as an anticancer compound modulating apoptosis [5], ROS production [6], cell proliferation [7], migration and invasion [8], and showing selective cytotoxic effect on several cancer cell types including pancreatic, renal, prostate, and breast cancers [5, 9–12]. Depending on the cell types, PL acts on various signaling pathways, including MAPK (p38/JNK) [13], nuclear factor kappa B (NF-κB) [14, 15], STAT3 [16], GSTP1 [17], and TrxR1 [18].

In this report, we studied the death potential of PL on two human retinoblastoma cell lines, WERI-Rb and Y79.

## RESULTS

### Piperlongumine induces cell death of WERI-Rb and Y79 retinoblastoma cell lines

**Figure 1:**
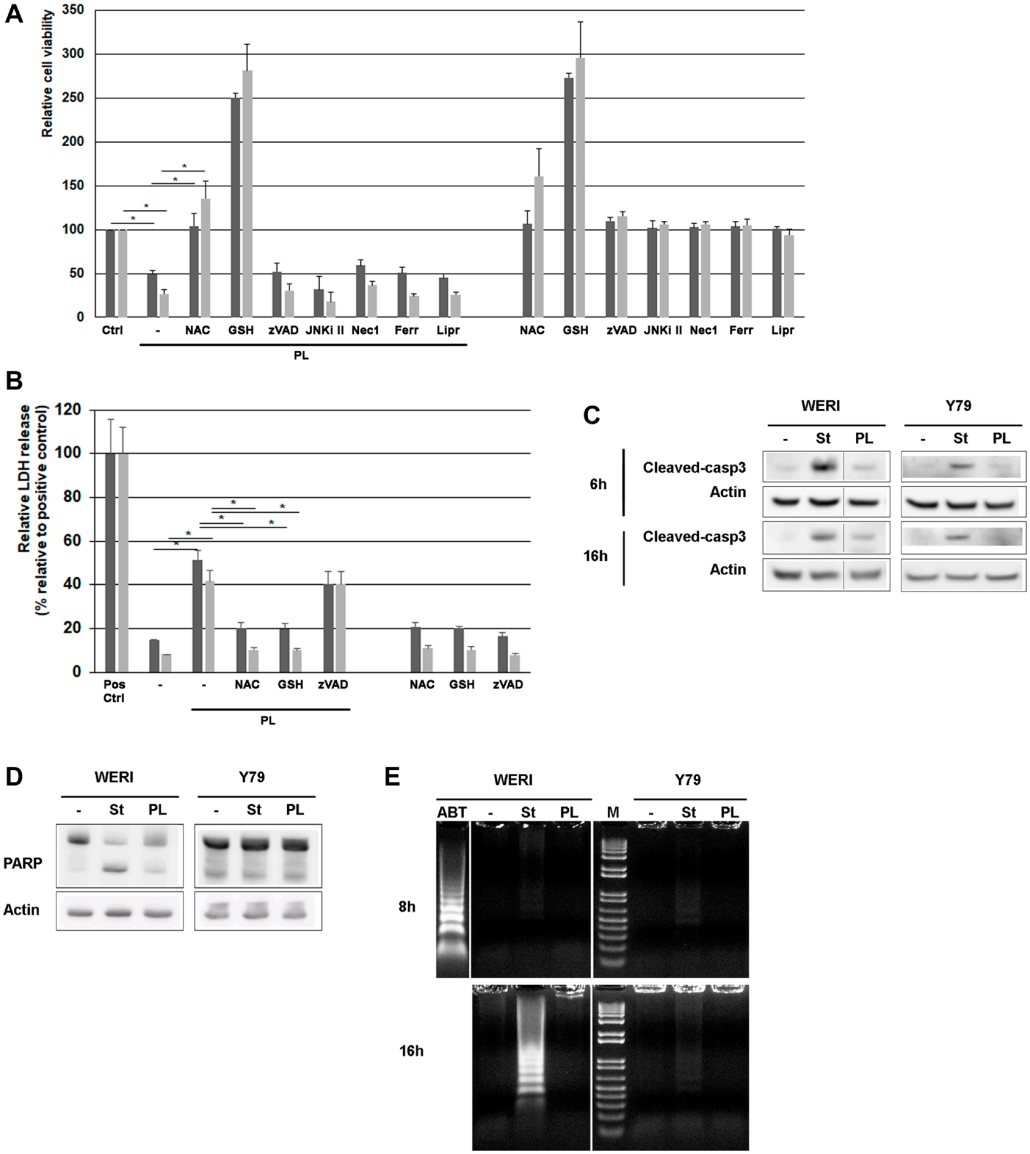
PL induces cell death in WERI-Rb and Y79 retinoblastoma cells. (**A**) WERI-Rb and Y79 cells were exposed to 10 μM PL for 24 h, and cell viability was determined using the Cell Counting Kit-8. Before PL treatment, cells were exposed for 1 hour to 3 μM NAC as wel as to 2 mM GSH, 10 μM zVAD (a broad-range caspase inhibitor), 10 μM JNKi II (a peptidic JNK inhibitor), 50 μM Nec-1 (a RIP-1 inhibitor), 1 μM Ferrostatin (Ferr) or 1 μM Liproxstatin (Lipr), both inhibiting ferroptosis. Dark grey column, WERI-Rb; grey column, Y79. Averaged media controls for multiple plates were set as 100% viability. Independent experiments, conducted in triplicate, have been repeated five times. ^*^ *P* < 0.005 by Student’s *t* test. (**B**) PL cytotoxicity was determined by measuring the activity of the LDH enzyme released by damaged cells. (**C**) WERI-Rb and Y79 cells were treated with 10 μM PL for 6 and 16 h and caspase-3 cleavage was determined by western blot experiments. Staurosporin (st) treatment (0.5 μM) is used as control. (**D**) WERI-Rb and Y79 cells were treated with 10 μM PL for 24 h and the cleavage of PARP, a downstream substrate of caspase-3, was determined by western blot experiment using an antibody that recognizes the full length and the cleaved protein (105 and 85 kDa). Staurosporin (st) treatment (0.5 μM) is used as control. (**E**) DNA laddering experiment. WERI-Rb and Y79 cells were exposed to 10 μM PL for 8 and 16 h and DNA fragmentation was investigated. M, Molecular weight marker. Treatment with 0.5 μM Staurosporin (st) and 1 μM ABT-737 (ABT) is used as control.

To assess the ability of PL to induce retinoblastoma cell death as a single agent, we exposed retinoblastoma cells to PL. As shown in Figure 1A, the proliferation assays revealed that the growth of both WERI-Rb and Y79 cancer cell lines was decreased by 2 and 3-fold respectively. LDH release experiments confirmed PL cytotoxicity against both cell lines (Figure 1B).

WERI-Rb treated with PL exhibited some classic signs of apoptosis like caspase-3 activation (Figure 1C) and downstream PARP cleavage (Figure 1D). However, DNA fragmentation could not be detected as indicated by DNA laddering (Figure 1E). In addition, other mechanisms than apoptosis should be involved in WERI-Rb killing as the broad-spectrum caspase inhibitor Z-VAD-FMK had no effect when added to WERI-Rb exposed to PL (Figure 1A).

Regarding Y79 cell death induced by PL, it appears to be caspase independent. Indeed, we were unable to measure any caspase-3 activation (Figure 1C), and downstream PARP cleavage (Figure 1D) in Y79 exposed to PL. No DNA fragmentation was either detected in Y79 (Figure 1E).

In addition to the use of the caspase inhibitor zVAD implicated in the apoptosis mechanism, we tested whether the necroptosis inhibitor Nec1, as well as the ferroptosis inhibitors Ferrostatin and Liproxstatin impacted the cell death triggered by PL. As shown in Figure 1A, none of these inhibitors protected retinoblastoma cells from death, suggesting that other mechanisms than necroptosis or ferroptosis are involved.

### Piperlongumine induces ROS production in WERI-Rb and Y79 retinoblastoma cell lines

Previous studies in several human cancer cell lines have reported that the cell death program engaged by PL included oxidative stress induction [6, 13, 19–22]. PL has been shown to inhibit Glutathione S-transferase pi 1 (GSTP1) by blocking its active site [17]. GSTP1, which is frequently overexpressed in tumors, has an important detoxifying function and provides cellular protection against free radical. Exposure of cancer cells to PL results therefore in increased ROS and decreased GSH.

**Figure 2:**
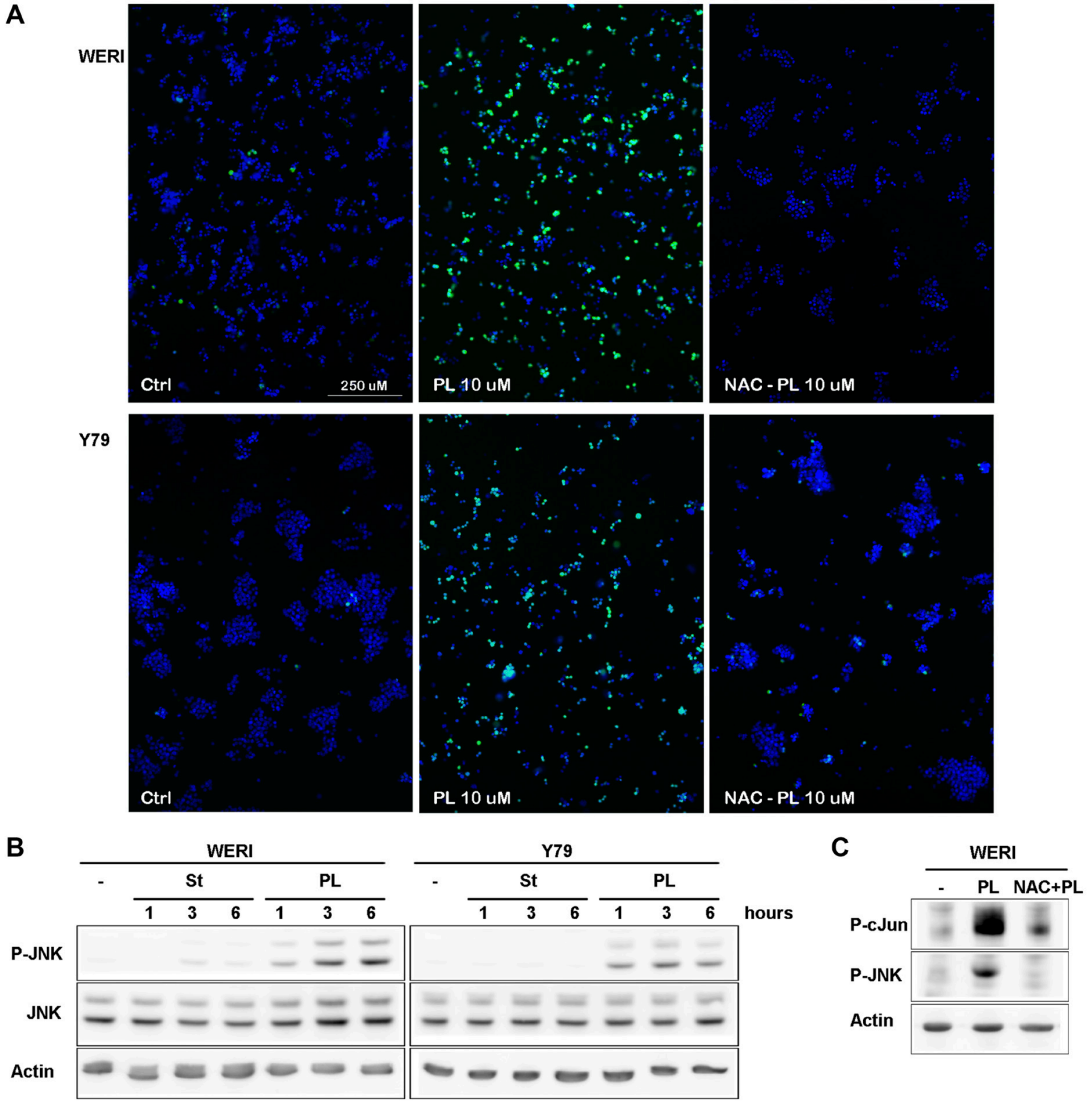
PL induces ROS production in WERI-Rb and Y79 followed by JNK activation. (**A**) WERI-Rb and Y79 were left untreated (ctrl) or treated with 10 μM PL during 3 h. Before PL treatment, cells were exposed for 1 h to 3 μM NAC. Cells were stained for 30 min with H2DCFDA and images were obtained with a fluorescent microscope. (**B**) WERI-Rb and Y79 were exposed to 10 μM PL for 1, 3 or 6 h. JNK activation was determined by western blot experiment using a P-JNK antibody. (**C**) WERI-Rb were exposed to 10 μM PL for 3 h without or with NAC. cJun phosphorylation was determined by western blot experiment using a P-cJun antibody.

After 3 h of treatment of WERI-Rb and Y79 cells with PL, increased levels of ROS production were readily observed (Figure 2A). As predicted, the PL-induced ROS accumulation was greatly reduced by the free-radical scavenger NAC, as well as by direct GSH addition (Figure 2A).

To further investigate the relationship between the ROS generation and PL-induced cell death, WERI-Rb and Y79 cells were exposed to PL in the presence or absence of NAC. NAC pretreatment for 1 hr completely blocked cell death (Figure 1A), suggesting that ROS production is critical for WERI-Rb and Y79 cancer cells death induced by PL.

ROS is known to activate JNK [23] and GSTP1 was shown to be a direct inhibitor of JNK [24]. We looked therefore at JNK activity in Y79 and WERI-Rb after PL treatment, and showed a tight correlation between ROS production and JNK activation in both cell lines (Figure 2B). In presence of NAC, JNK activity was greatly reduced (Figure 2C). However, blocking JNK activity using the JNK inhibitor peptide (JNKI) [25], we were unable to protect WERI-Rb and Y79 cells from death, suggesting that JNK is located in the downstream part of the death pathway (Figure 1A).

### PL modulates the transcript level of cell cycle-regulatory factors

**Figure 3:**
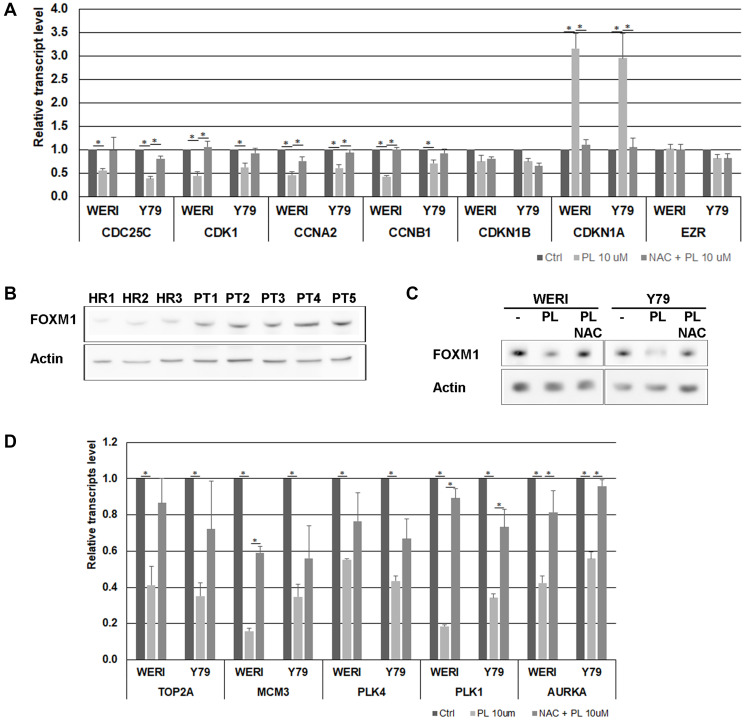
Effects of PL on cell cycle-regulatory factors in WERI-Rb and Y79 cells. (**A**) The expression of *CDC25C, CDK1, CCNA2, CCNB1, CDKN1A* and *CDKN1B* was measured at mRNA levels using real time RT-PCR. *EZR* transcript, encoding a cytoplasmic peripheral membrane protein, was used as a negative control and *GAPDH* was used as an internal control gene. Data represent the mean ± SEM (*n* = 3). ^*^ *P* < 0.05 by Student’s *t* test. (**B**) The content of FOXM1 was determined in three healthy mouse retina (HR) and in five primary mouse retinoblastoma (PT) by western blot analysis. (**C**) WERI-Rb and Y79 were exposed to 10 μM PL for 24 h without or with 1 h pre-treatment with 3 μM NAC. FOXM1 protein level was determined by western blot experiment. (**D**) The expression level of FOXM1 target genes was determined by real time RT-PCR. *GAPDH* was used as an internal control gene. Data represent the mean ± SEM (*n* = 3). ^*^ *P* < 0.05 by Student’s *t* test.

PL has been shown to modulate the expression of cell cycle factors, such as cyclins and CDKs [26]. To determine the effects of PL on the transcription of cell cycle-associated genes in WERI-Rb and Y79 cells, real-time RT-PCR were performed. The results showed that the expression of CCNA2, CCNB1, CDC25C and CDK1 mRNA was significantly decreased in cells treated with PL (Figure 3A). It is also well established that CDKs activity and cell cycle progression can be attenuated by CDKN1A [27]. Real-time PCR experiments displayed that PL increases CDKN1A mRNA content in WERI-Rb and Y79 cells, while no variation in the CDKN1B transcript was observed (Figure 3A).

Our results suggest therefore that PL impacts the cell cycle by decreasing the level of cyclins and CDK1 and by increasing the content of the CDKs inhibitor CDKN1A. All these variations triggered by PL were acting through ROS accumulation, as they were almost completely abolished when cells were pre-treated with NAC (Figure 3A).

### PL modulates the expression of FOXM1

Forkhead Box M1 (FOXM1) is a transcription factor of the members of the forkhead family of proteins, which plays a critical regulatory role in the cell cycle progression by targeting several cell cycle genes, such as *CCNA2*, *CCND1*, *SKP2* and *CDC25A* during the G1/S transition phase; *TOP2A*, *MCM3* and *PLK4* during the S phase; *CCNB1*, *CCNB2*, *CDK1*, *AURKA*, *Survivin*, *PLK1*, *PRC1* during the G2/S transition phase and mitosis [28]. FOXM1 has also a transcriptional inhibitory effect on *CDKN1A*. FOXM1 has been described as a promoter of cell proliferation in a variety of tumors [29], and FOXM1 content was shown to be downregulated in breast and pancreatic cancer cell lines following PL exposure [30]. Microarray analysis have detected an upregulation of *FOXM1* in human retinoblastoma [31], and in retinoblastoma Y79 cells, *FOXM1* depletion has been shown to affect cell invasive capacity by targeting *MMP2* [32]. We observed that *FOXM1* is overexpressed in retinoblastoma tumors isolated from an SV40-Tag mouse model (Figure 3B), and that *FOXM1* content was downregulated following WERI-Rb and Y79 cell exposure to PL (Figure 3C). We investigated the expression modulation of FOXM1 target genes by real-time RT-PCR and observed that the expression of these different genes was significantly decreased in cells treated with PL (Figure 3D). These results suggest a potential role of FOXM1 in the cell death induced by PL through cell cycle dysregulation.

## DISCUSSION

The aim of our study was to determine the cytotoxicity of PL against retinoblastoma cell lines. The main known mechanism used by PL to induce cell death is the excessive production of cellular ROS [20, 33]. We observed that PL increased ROS accumulation in WERI-Rb and Y79 cells (Figure 3A), while pre-treatment of cells with NAC attenuated this accumulation and protected cells from death. It is well known that cancer cells have developed the capacity to reprogram their energy metabolism to survive in a rough environment [34, 35]. This adaptation has for consequence the increase of ROS production. Various studies have shown that the impact of ROS in cancer cells is dependent on the stage of the tumor. In early stages, the accumulation of intracellular ROS promotes oxidative DNA damage and mutations into pro-oncogenes and tumor suppressor genes [36]. In later advanced stages, excessive levels of ROS have been shown to increase the sensitivity of these cancer cells to cell death, making them more vulnerable to additional ROS enhancement [36]. In our study, the exposure of WERI-Rb et Y79 to PL induced an overproduction of ROS leading to their death.

ROS has been described to mediate various programmed cell death including apoptosis, autophagy, pyroptosis, necrosis, and ferroptosis [37]. When considering markers of apoptosis after exposure to PL, we demonstrated that apoptosis was only weakly triggered in WERI-Rb and not involved in Y79 cell death. Caspase-3 activation by PL was very faint in WERI-Rb and undetectable in Y79, as observed by direct assays (Figure 1C), or indirectly by examining caspase-3 substrate cleavage (PARP) (Figure 1D). We also investigated DNA fragmentation following PL treatment, even if absence of DNA laddering may not be a convincible evidence for the inexistence of apoptosis. Figure 1E showed that PL did not induce DNA fragmentation neither in WERI-Rb, nor in Y79.

In addition to massive ROS production, studies about the effect of PL on tumors have depicted multiple mechanisms. Depending on the cell types, PL has an impact on various signaling pathways, including MAPK (p38/JNK) [13], nuclear factor kappa B (NF-κB) [14, 15], STAT3 [16], GSTP1 [17], and TrxR1 [18]. We assessed the potential role of necroptosis and ferroptosis in WERI-Rb and Y79 cell death triggered by PL using specific inhibitors (Figure 1A). None of them showed any protective effect. We also investigated specific markers of these two programmed cell deaths, i.e., RIPK1 and RIPK3 phosphorylation for necroptosis, and lipid peroxidation for ferroptosis. None of them were modulated following PL treatment (data not shown).

We also examined the autophagy process, but we were unable to observe any LC3II or p62 modulation in WERI-Rb and Y79 following PL treatment (data not shown).

Previous studies have established the effect of ROS on cell cycle progression by modulating cell cycle regulators, such as cyclins and cyclin dependent kinase inhibitor [38, 39]. We determined that PL disturbed the expression of various factors involved in the cell cycle progression including *CDK1*, *CDC25C*, *CDKN1A* (Figure 3A). Several of these genes are known to be regulated by *FOXM1* at transcriptional level. We found that FoxM1 protein expression was increased in primary mouse retinoblastoma (Figure 3B) and PL exposure of WERI-Rb and Y79 induced a decrease in FOXM1 protein content (Figure 3C).

As a master regulator of the cell cycle, the transcription factor FOXM1 is required for cell proliferation of normal cells, and it is an important factor in various types of cancer [28, 29, 40]. The oncogenic potential of FOXM1 is based on its faculty to regulate the expression of target genes involved in cell cycle transition, cell proliferation, chromosome stability, stem cell renewal, and later phases of tumorigenesis. Indeed, studies aimed at FOXM1 inhibition in cancer cells have observed a decrease in cell proliferation and migration, metastasis, angiogenesis, EMT, and drug resistance, demonstrating the implication of FOXM1 in these different processes [40–45]. FOXM1 is therefore a potential therapeutic target in cancer therapy potentialy inhibited by PL. As FOXM1 is involved in various signaling pathways -other than cell cycle- that control many key cancer properties, it would be interesting to determine whether PL has a effect on these pathways using animal model developping retinoblastoma.

Our study reports that PL induces retinoblastoma cells death through the accumulation of ROS resulting in cellular oxidative stress. A potential role of FOXM1 in this cell death process has to be verified using inhibitors or following FOXM1 overexpression. These data provide *in vitro* evidence that PL could serve as a potential anticancer molecule in retinoblastoma treatment.

## MATERIALS AND METHODS

### Chemicals

Piperlongumine (PL), N-acetyl-L-cysteine (NAC), Necrostatin-1 (Nec1), Ferrostatin-1, Liproxstatin, Glutathione (GSH), Ciclopirox (CPX) were purchased from Sigma-Aldrich (Sigma, St. Louis, USA), zVAD was from Promega (Promega, Madison, WI, USA).

The primary antibodies and their dilutions used for western blotting experiments were as followed, beta-actin (A5441, 1:1000, Sigma, St. Louis, USA), PARP (sc-7150, 1:1000, Santa Cruz Biotechnology, Santa Cruz, USA), cleaved-caspase 3 (9661, 1:1000, Cell Signaling, Danvers), P-JNK (4668, 1:1000, Cell Signaling, Danvers), JNK (sc-571, 1:1000, Santa Cruz Biotechnology, Santa Cruz, USA), P-c-Jun (9261, 1:1000, Cell Signaling, Danvers), OPA1 (BD Bioscience 612606), PRC1 (1:1000, Protein Tech Group, Chicago, USA), FOXM1 (sc-376471, 1:1000, Santa Cruz Biotechnology, Santa Cruz, USA), GPX4 (ab125066, 1:1000, Abcam, Cambridge, UK).

The secondary antibodies used for Western blotting experiments were as followed: ECL anti-rabbit IgG horseradish peroxidase linked, ECL anti-mouse IgG horseradish peroxidase linked (Amersham Biosciences, Otelfingen, Switzerland).

### WERI-Rb and Y79 cell cultures

WERI-Rb and Y79 cell lines were obtained from ATCC (Manassas, VA, USA) and were cultured in RPMI 1640 medium supplemented with 100 μg/ml streptomycin, 100 units/ml penicillin, 1 mM sodium pyruvate, 2 mM glutamine and 10% fetal calf serum (20% for Y79).

### Cytotoxicity assays and LDH release

Cells were seeded at a density of 10,000 cells per well in 96-well plates, incubated overnight in 10% FBS/RPMI (20% for Y79), then treated for varying lengths of time with various chemical compounds. Following treatment, drug toxicity was measured using the Cell Counting Kit-8 developed by Sigma-Aldrich and the CyQUANT LDH Cytotoxicity Assay Kits from ThemoFischer Scientific (Waltham, MA, USA) using a microplate reader (Bio-Tek Instruments, Winooski, VT, USA).

Mean values were obtained from five independent experiments, each conducted in triplicate.

### ROS production and microscopy

Following exposure to PL, cells were stained with 10 μM of the dye dihydrodichlorofluorescein-diacetate (H2DCFDA, Invitrogen Inc., Eugene, OR, USA) in 1 ml media to measure intracellular hydrogen peroxide. Cells were stained for 30 min at 37°C.

Fluorescence microscopy was performed on a Leica DM6000B Microscope, equipped with a Leica DFC365 FX digital camera. Images were captured using the Leica Application Suite (LAS-AF) microscope software. Representative pictures were taken using a 40x/0,85 Leica HC PL-APOCHROMAT objective.

### DNA laddering

After cells have been exposed to chemical compounds, study of DNA laddering was conducted as previously described [46].

### Whole cell lysates

Cells were washed once in cold Phosphate Buffered Saline (PBS) and recovered by centrifugation. Briefly, cell pellets were dislodged into cold lysis buffer (20 mM Tris-acetate pH 7.0, 0.27M sucrose, 1 mM EDTA, 1 mM EGTA, 50 mM sodium fluoride, 1%Triton X-100, 10 mM β-glycero-phosphate, 1 mM DTT, 10 mM p-nitrophenyl-phosphate, and antiproteases), and centrifuged at 15,000 rpm for 20 minutes. Supernatants were recovered and stored at –70°C until use. Total protein in cell lysates was quantified using the BCA Protein Assay according to the manufacturer (Life Technologies, Carlsbad, CA, USA).

### Western blotting experiments

Equal quantities of total protein lysates (40 ug per well) were resolved by 8–15% SDS-polyacrylamide gel electrophoresis and electrotransferred onto polyvinylidene difluoride membranes. Nonspecific protein binding was blocked by incubating the membranes with a blocking solution (1x TBS, 0.1% Tween 20, 5% nonfat dried milk powder) for 1 h at room temperature. The blots were then probed overnight with primary antibodies. The immune complex was detected by using a peroxidase-conjugated secondary antibody and the chemioluminescent detection kit according to the manufacturer’s specifications (EMD Millipore EMD Millipore, Merck KGaA, Darmstadt, Germany). FUJIFILM Multi Gauge software was used for densitometric analysis.

### RNA isolation

Total RNA was extracted from treated cells using the TRIzol reagent (Invitrogen AG, Basel, Switzerland) and following the manufacturer’s instructions. Both quantity and quality of RNA were determined on a ND-1000 spectrophotometer (NanoDrop technologies, Inc., Wilmington, DE).

### Reverse transcription and quantitative PCR

cDNA synthesis was performed using 2 μg of total RNA in 20 μl reaction volume. This was done using an oligo dT primer according to the manufacturer’s manual (Affinity Script; Stratagene; Agilent technologies SA, Morges, Switzerland). For quantitative PCR, cDNA obtained from 50 ng original total RNA was used for PCR amplification using the 2× brilliant SYBR Green QPCR Master Mix (Agilent) with 250 nM of forward and reverse primer, designed to span an intron of the target gene (Table 1). Real-time PCR was performed in triplicate in a Mx3000PTM system (Agilent) with the following cycling conditions: 40 cycles of denaturation at 95°C for 30 sec, annealing at 59°C for 30 sec, and extension at 72°C for 30 sec. Quantitative values were obtained by the cycle number (Ct value) reflecting the point at which fluorescence starts to increase above background at a fixed threshold level. Values obtained for the target genes were normalized with the housekeeping gene *Gapdh.*

**Table 1:** Primers sequence (5′–3′)

CDC25C-F	TCTCCTGGTGAGAATTCGAAGA
CDC25C-R	GAGGCAACGTTTTGGGGTTC
CDK1-F	TGGAAATTGAGCGGAGAGCG
CDK1R	TGGCTACCACTTGACCTGTAG
CCNA2-F	CTGCGTTCACCATTCATGTGG
CCNA2-R	ACACTCACTGGCTTTTCATCTTC
CCNB1-F	CCTCTCCAAGCCCAATGGAA
CCNB1-R	ACTTCCCGACCCAGTAGGTA
CDKN1B-F	ACCTGCAACCGACGATTCTT
CDKN1B-R	GTCCATTCCATGAAGTCAGCG
CDKN1A-F	AGCAGAGGAAGACCATGTGG
CDKN1A-R	TTCCAGGACTGCAGGCTTCC
EZR-F	AAGGATTTCCTACCTGGCTG
EZR-R	GGCAGTAGATCTCGTCGCGA
MCM3-F	TGATGCTACCTATGCCAAGC
MCM3-R	GTCTTCTTAGTAGCAGGACAG
PLK4-F	TTTGCTGGTGTCTACAGAGC
PLK4-R	CTCCATTATGGCACATTTCTA
PLK1-F	TTTCGAGGACAACGACTTCG
PLK1-R	CATTCAGGAAAAGGTTGCCC
AURKA-F	GGAGGAACTGGCATCAAAAC
AURKA-R	TAAGAGCCAGAATAAACTTGCT

### Animal handling

The SV40-Tag (C57BL/6) mice [47] were a gift from Dr. Joan O’Brien. The animals were maintained and euthanized in accordance with the ARVO Statement for the Use of Animals in Ophthalmic and Vision Research and were approved by the local Committee Office on Use and Care of Animals in Research of the State of Valais, Sion, Switzerland.

### Statistical analysis

All results were expressed as means ± SEM of the indicated number of experiments. For statistical analysis, Student’s *t*-test was performed and *P* values of less than 0.05 were considered to be statistically significant.
